# The effects of repetitive transcranial magnetic stimulation in an animal model of tinnitus

**DOI:** 10.1038/srep38234

**Published:** 2016-12-01

**Authors:** Wilhelmina H. A. M. Mulders, Vanessa Vooys, Kalina Makowiecki, Alex D. Tang, Jennifer Rodger

**Affiliations:** 1 The Auditory Laboratory, School of Anatomy, Physiology and Human Biology, The University of Western Australia, 35 Stirling Highway, Crawley WA 6009, Australia; 2 School of Animal Biology, The University of Western Australia, 35 Stirling Highway, Crawley WA 6009, Australia

## Abstract

Tinnitus (phantom auditory perception associated with hearing loss) can seriously affect wellbeing. Its neural substrate is unknown however it has been linked with abnormal activity in auditory pathways. Though no cure currently exists, repetitive transcranial magnetic stimulation (rTMS) has been shown to reduce tinnitus in some patients, possibly via induction of cortical plasticity involving brain derived neurotrophic factor (BDNF). We examined whether low intensity rTMS (LI-rTMS) alleviates signs of tinnitus in a guinea pig model and whether this involves changes in BDNF expression and hyperactivity in inferior colliculus. Acoustic trauma was used to evoke hearing loss, central hyperactivity and tinnitus. When animals developed tinnitus, treatment commenced (10 sessions of 10 minutes 1 Hz LI-rTMS or sham over auditory cortex over 14 days). After treatment ceased animals were tested for tinnitus, underwent single-neuron recordings in inferior colliculus to assess hyperactivity and samples from cortex and inferior colliculus were taken for BDNF ELISA. Analysis revealed a significant reduction of tinnitus after LI-rTMS compared to sham, without a statistical significant effect on BDNF levels or hyperactivity. This suggests that LI-rTMS alleviates behavioural signs of tinnitus by a mechanism independent of inferior colliculus hyperactivity and BDNF levels and opens novel therapeutic avenues for tinnitus treatment.

Tinnitus is a common phantom auditory perception, affecting 10–15% of the population, with approximately 2% reporting debilitating symptoms^1^,^2^, such as insomnia, emotional difficulties and depression^3^,^4^. Despite its high prevalence and economic and social costs, there is still no universal cure. Tinnitus is strongly correlated with hearing loss^5^,^6^. Current theories suggest that cochlear insult leads to tinnitus by triggering plasticity in the auditory pathway. Human and animal studies suggest these plastic changes involve increased spontaneous activity, changes in temporal patterns of activity and/or reorganization of tonotopic maps in the auditory pathway^7^,^8^,^9^,^10^,^11^. Targeting neural activity and plasticity in specific auditory brain structures therefore has potential for treating tinnitus.

One therapeutic approach is to modulate brain activity using non-invasive stimulation techniques such as repetitive transcranial magnetic stimulation (rTMS). rTMS induces a focalized electric field in brain tissue by electromagnetic induction. This depolarises neurons^12^ and induces different forms of neural plasticity^13^. rTMS induced plasticity is thought to be frequency dependent, with low frequencies (<1 Hz) being inhibitory and high frequencies (>5 Hz) excitatory^14^. Plastic changes are also observed following both low and high intensity rTMS^15^,^16^,^17^,^18^,^19^,^20^,^21^. Thus, by varying frequency, intensity and the targeted brain region, it may be possible to treat specific neurological conditions including tinnitus^14^.

Several studies have investigated rTMS application in tinnitus patients and some show improvements/benefits that persist beyond the stimulation period^22^,^23^,^24^,^25^,^26^,^27^. The high variability in efficacy and duration of symptom suppression between studies is presumably associated with the widely differing parameters applied in each case: high or low frequency stimulation^28^, applied over auditory or prefrontal cortex, one-sided or bilaterally^22^,^29^. The lack of parameter consistency highlights our poor understanding of the mechanisms of plasticity induced by rTMS. Animal studies may establish the underlying biological mechanisms of rTMS in tinnitus and permit a more directed approach in the clinical setting.

We have developed a guinea pig model of tinnitus, using unilateral exposure to a loud tone to damage the cochlea, leading to a small hearing loss and development of increased spontaneous firing rates (hyperactivity) of central auditory neurons^10^,^30^,^31^. Additionally, we have demonstrated behavioural evidence for tinnitus in this model^32^,^33^ in agreement with other similar models^34^,^35^,^36^,^37^. Here, we use our model to investigate whether rTMS can reduce behavioural evidence of tinnitus and the associated hyperactivity in inferior colliculus (IC). Upon behavioural evidence of tinnitus, we commenced treatment (10 minutes of 1 Hz low intensity rTMS (LI-rTMS) or sham stimulation over auditory cortex contralateral to the affected cochlea for 10 daily sessions over 14 days) using a custom-built coil^38^,^39^. This coil approximates the coil to brain-size ratio used in humans providing improved focality^40^. We also investigated a possible mechanism by which LI-rTMS could influence tinnitus, by measuring hyperactivity in IC and brain-derived neurotrophic factor (BDNF) levels in IC and auditory cortex. BDNF plays important roles in neural plasticity^41^ and was upregulated in other neural systems in rodents following rTMS^16^,^42^.

## Results

### Establishing behavioural evidence of tinnitus

**Figure 1:**
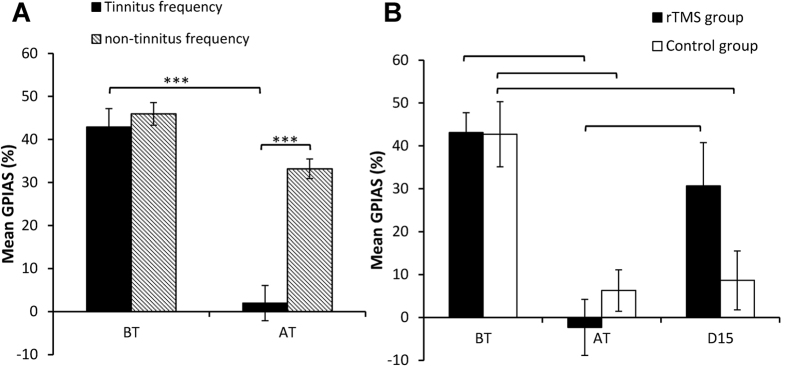
Effect of rTMS versus sham treatment in the 12 animals that developed the behavioural signs of tinnitus. (**A)** Shows the percentage GPIAS at the tinnitus frequency (n = 12; for the two animals that showed a GPIAS deficit at both frequencies tested, the mean percentage suppression at both frequencies was calculated) and non-tinnitus frequency (n = 10; this excludes the two animals that developed tinnitus at both background frequencies) before (BT) and after trauma (AT) in all animals that developed signs of tinnitus. (**B**) Shows the percentage GPIAS at the frequency at which the animal developed tinnitus (for the two animals that showed a GPIAS deficit at both frequencies tested, the mean percentage suppression at both frequencies was calculated). Graph shows the data before acoustic trauma (BT), in the week the animals developed a GPIAS deficit (after acoustic trauma, AT), and at day 15 (D15). Treatment was applied day 1 to 5 (Monday to Friday) and day 7 to 12 (Monday to Friday). D15 was the Monday after the treatment had finished and the final electrophysiological experiment took place on either D15 or D16.

All animals passed the (pre-pulse inhibition) PPI and (gap pre-pulse inhibition of acoustic startle) GPIAS test twice before they were exposed to an acoustic trauma (10 kHz, 124 dB SPL, 2 hours). Six of these animals (33%) did not develop a GPIAS deficit; defined as passing PPI and failing GPIAS twice in one week, within 12 weeks after acoustic trauma and did therefore not proceed to LI-rTMS or sham treatment and are not further discussed in this manuscript. The fact that not all animals developed signs of tinnitus is in agreement with other animal models^43^,^44^,^45^ and with observations in humans that not all individuals with a hearing loss develop tinnitus^1^,^46^. The remaining 12 animals (67%) did develop a GPIAS deficit between 2 and 9 weeks (mean 5.2 ± 0.5 weeks) following acoustic trauma. In 5 animals the GPIAS deficit appeared at the background noise of 14 kHz, in another 5 animals at the background noise of 8 kHz and in the final 2 animals the deficit occurred at both frequencies. These animals were therefore deemed to show behavioural evidence of tinnitus. Figure 1A shows the percentage GPIAS for these 12 animals at the frequency at which they developed tinnitus and at the frequency at which they did not develop tinnitus before acoustic trauma (BT) and after acoustic trauma (AT). One-way ANOVA with repeated measures factor time (percentage GPIAS before trauma, after trauma) between-subjects factor (tinnitus frequency and non-tinnitus frequency) showed a statistically interaction between time and frequency (F (1, 40) = 15.34, p = 0.0003). The significant interaction was followed by Sidak corrected post-hoc tests and this revealed a significant decrease in percentage GPIAS at the tinnitus frequency after acoustic trauma (p < 0.0001) and a significant difference after acoustic trauma between the tinnitus frequency and the non-tinnitus frequency (p < 0.001). Importantly no difference was found at the non-tinnitus frequency before and after acoustic trauma, demonstrating that GPIAS suppression was robust. When tinnitus was confirmed, animals were then randomly assigned to rTMS or sham treatment (10 minutes daily for 10 working days).

An ANOVA with repeated measures factor time (percentage GPIAS before trauma, after trauma and after rTMS treatment) between-subjects factor treatment condition (LI-rTMS and sham) was performed. This showed a statistically significant effect of time on percentage GPIAS (F(2, 20) = 29.93, p < 0.0001; Fig. 1B). A significant interaction between time and treatment condition was found (F (2, 20) = 4.392, p = 0.0262), showing that GPIAS changed differently dependent on treatment, but there was no significant main effect of treatment condition combined across time-points (F (1, 10) = 0.3486, P = 0.5680). The significant interaction was followed up by Sidak corrected post-hoc tests and this revealed a significant decrease in percentage GPIAS across groups after acoustic trauma (p < 0.001), confirming the development of tinnitus after acoustic trauma. Following the 10 weekdays of LI-rTMS treatment, percentage GPIAS significantly increased (p < 0.001) compared to the post-trauma response, returning to pre-trauma levels. By contrast, sham treatment did not rescue percentage GPIAS, with responses remaining significantly decreased compared to pre-trauma values (p < 0.001). These data suggest that LI-rTMS but not sham treatment reversed the behavioural signs of tinnitus.

### CAP audiograms

**Figure 2:**
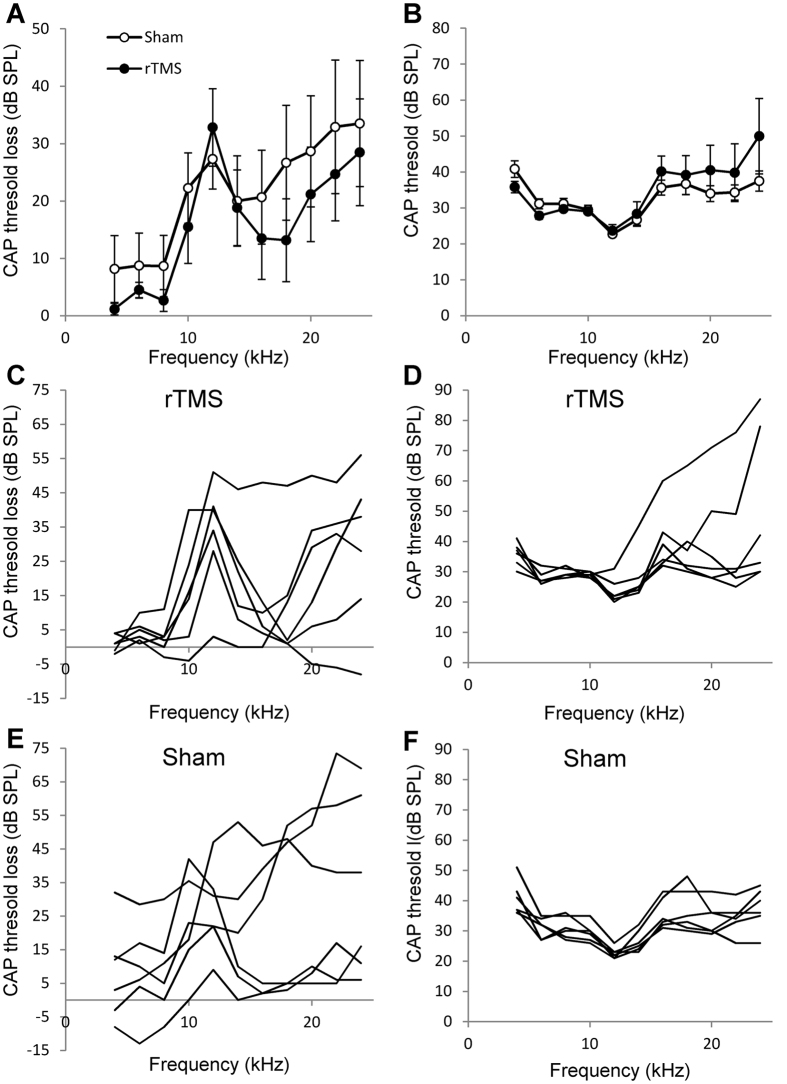
Audiogram data from the animals in study 2 that developed tinnitus. **(A**) shows mean CAP threshold loss in the left ear at the time of the final single neuron recording experiment (varying between 5 and 12 weeks after acoustic trauma) for the 6 rTMS and 6 sham treated animals. The CAP threshold losses in the individual animals are shown in (**C**) (rTMS treated animals) and 2E (sham treated animals). No statistical significant differences were found between the rTMS or sham treated animals (one way multivariate ANOVA). (**B**) shows the mean peripheral thresholds in the right ear measured during the final electrophysiological experiment and (**D**,**F**) show the individual right ear audiograms in the rTMS and sham treated group, respectively. No statistically significant difference was found between the right ear thresholds of rTMS and sham treated animals (one way multivariate ANOVA).

To confirm that hearing loss was present in the acoustic trauma ear and that LI-rTMS did not affect normal hearing in the contralateral ear, all animals underwent (compound action potential) CAP recordings in both ears after GPIAS and PPI behavioural testing (Fig. 2). Acoustic trauma resulted in a small to moderate ipsilateral permanent CAP threshold loss at frequencies >10 kHz (Fig. 2A), a result consistent with previous studies from our laboratory^10^,^47^,^48^ and from others^11^,^49^. Additionally, in many animals there was hearing loss at 20–24 kHz (Fig. 2C,E), which is also consistent with previous studies^50^,^51^,^52^. We ran a two-way ANOVA to compare treatment conditions (LI-rTMS vs. sham) with frequency as a repeated measures factor. Mean CAP threshold loss in the left ear at the time of the final single neuron recording experiment (varying between 5 and 12 weeks after acoustic trauma) was not significantly different between LI-rTMS and sham treated animals (F (1, 10) = 0.490; p = 0.5; Fig. 2A), although there was high variability between animals (Fig. 2C,E).

A similar analysis for the mean peripheral thresholds in the right ear, comparing LI-rTMS treated animals to sham, showed no significant effect of LI-rTMS (Fig. 2B; individual data in 2D and F; F (1, 110) = 1.706, p = 0.1942).

### Single neuron recordings of spontaneous activity

**Figure 3:**
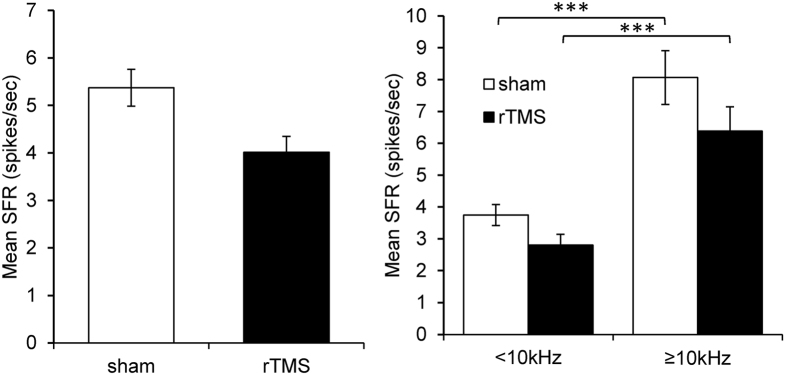
Spontaneous firing rate in the rTMS treated animals and sham treated animals. (**A**) shows that although rTMS treatment resulted in an apparent decrease of spontaneous firing rate a Mann-Whitney test showed this change was not statistically significant (p = 0.1258). (**B**) shows that the spontaneous activity is significantly higher in the IC frequency region ≥ 10 kHz compared to the frequency region <10 kHz in both sham and rTMS treated animals (Kruskall-Wallis followed by Dunn’s multiple comparisons tests; p < 0.001), but no statistical difference was observed in either subpopulation. ***p < 0.001.

Spontaneous firing rates were obtained from 552 neurons from the 6 sham treated animals (varying between 89 and 95 neurons per animal) and from 531 neurons from the 6 LI-rTMS treated animals (varying between 77 and 93 neurons per animal. Although LI-rTMS treatment decreased spontaneous firing rates, a Mann-Whitney test showed this decrease was not statistically significant (Mann-Whitney U = 138804; p = 0.1258; Fig. 3A). In addition since it is known that the spontaneous activity is predominantly increased in the frequency region of hearing loss^10^,^33^,^48^, we divided the neurons into two groups based on CF (<10 kHz and ≥10 kHz), representing the frequency range of no peripheral hearing loss versus the frequency range of hearing loss, respectively (see audiograms in Fig. 2). The results are shown in Fig. 3B. The frequency region in IC ≥10 kHz shows higher average spontaneous firing rates compared to the <10 kHz region in both sham and rTMS treated groups (Kruskall-Wallis with Dunn’s post-tests p < 0.001 for both groups). No statistically significant differences were found between sham and rTMS treatment in either frequency region.

### BDNF

**Figure 4:**
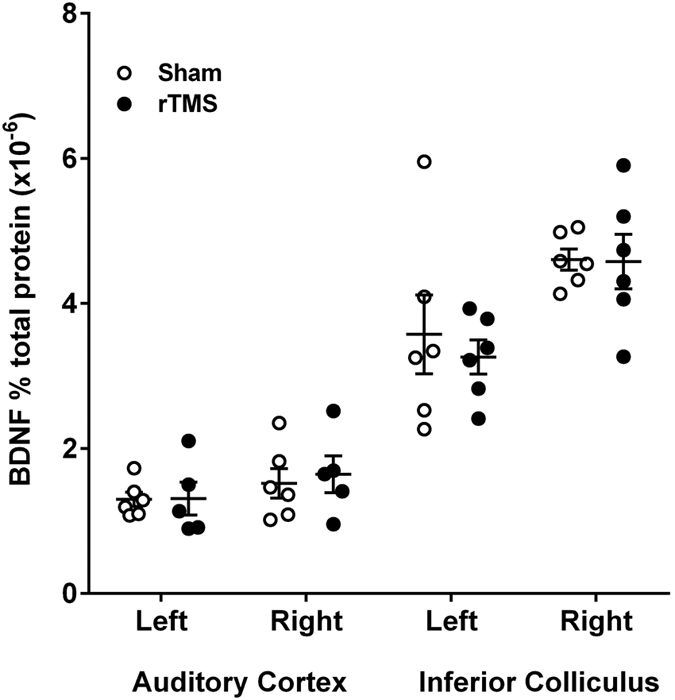
BDNF ELISA data in the rTMS treated animals and sham treated animals. Scatterplot showing individual data (open and closed circles) and mean ± SEM of BDNF concentrations in the right and left auditory cortex and inferior colliculi. For inferior colliculus, no significant difference was found between rTMS and sham, though there was a significant difference between left and right within-subjects independent of treatment. In auditory cortex, no significant effects were found.

To investigate whether BDNF might play a role in the effect of LI-rTMS in our animal model of tinnitus, BDNF concentrations in the right and left auditory cortex and inferior colliculi were measured using ELISA (Fig. 4). There was a significant difference between left and right IC within-subjects (F (1,10) = 8.93, p = 0.01), but this was not dependent on treatment, as there was no significant interaction between side and stimulation condition (F (1,10) = 0.13, p = 0.72) and no significant difference between LI-rTMS and sham (across sides, F (1,10) = 0.28, p = 0.61). This difference between sides was therefore most likely due to the acoustic trauma.

In auditory cortex, LI-rTMS had no significant effect on BDNF concentration as there was no significant difference between LI-rTMS and sham (F (1,9) = 0.07, p = 0.79), or between stimulated and non-stimulated side within the same animal (F (1,9) = 5.06, p = 0.05), and no significant interaction (F (1,9) = 0.22, p = 0.65).

## Discussion

The present data show in a guinea pig model that low frequency LI-rTMS over the auditory cortex can alleviate behavioural signs of tinnitus. In this animal model tinnitus was caused by a unilateral acoustic trauma and LI-rTMS was applied to the auditory cortex on the contralateral side. Single neuron recordings showed a small reduction of the increased spontaneous firing rates in the IC after LI-rTMS but this reduction was not statistically significant. Analyses of BDNF expression suggest that BDNF signalling mechanisms are not strongly linked to LI-rTMS-induced alleviation of behavioural signs of tinnitus, but BDNF levels may be affected by acoustic trauma.

### Effects on behavioural evidence of tinnitus

GPIAS was used to behaviourally assess tinnitus in the animals. GPIAS measures the animal’s ability to detect a silent gap embedded in background noise. If an animal is perceiving tinnitus at a frequency similar to the background noise, then it is thought that tinnitus will “fill in” the silent gap. In addition, PPI was used to exclude the possibility that an animal would fail the GPIAS test due to hearing loss. This test has been validated by many studies^37^,^43^,^53^ and has been correlated with operant methods of assessing tinnitus in animals^37^.

Our results showed that the low frequency LI-rTMS applied over the auditory cortex for short sessions over a period of two weeks caused a significant change in the GPIAS percentage, suggesting attenuation of the perception of tinnitus. Tinnitus is associated with abnormal levels of activity in the central auditory system in human tinnitus patients^9^,^54^,^55^,^56^. One of the main mechanisms of rTMS delivered at 1 Hz is believed to involve long-term depression (LTD). In agreement, human studies, using functional imaging, have shown that daily 1 Hz rTMS can reduce overall activity in the auditory cortex^22^,^57^. However, it should be noted that as it is unknown what neural elements of the cortex are targeted by rTMS, it is uncertain whether the decreased neural activity is the result of decreased excitation or increased inhibition^45^,^58^.

The beneficial effect of rTMS treatment observed in our data is reflective of rTMS studies in human tinnitus patients, which have shown alleviation of tinnitus symptoms in some, but not all patients using high intensity rTMS^22^,^23^,^59^,^60^,^61^. These human studies show mixed efficacy and varying duration of suppression of the tinnitus symptoms but comparison between them is complicated by the fact that a variety of parameters are applied in the different studies, such as using either high or low frequency stimulation^28^, applied either over the auditory or prefrontal cortex, one-sided or bilaterally^22^,^29^. One important difference between human studies and our animal model is that the animals were treated as soon as tinnitus developed whereas human subjects treated with rTMS generally have chronic tinnitus of long standing. Date from our animal model suggests that there are ongoing changes throughout tinnitus development and therefore effects of treatment applied early may differ from effects of the same treatment at a later stage^10^,^30^,^32^,^62^.

Furthermore, tinnitus is measured in a different way in human and animal studies, which could potentially affect outcomes. Human studies rely on self-reporting and other associated measures such as tinnitus distress, loudness and intrusiveness, whereas animal studies use behavioural tests such as the GPIAS test used in the present study. Nonetheless it has been shown that human tinnitus subjects also show deficits in gap pre-pulse inhibition, in line with the animal behaviour test used in the present study^63^. In addition, tinnitus induction in our animal model is triggered by an acute acoustic trauma which will only be the case for a subset of tinnitus patients. Translational human studies will be necessary to elucidate whether the beneficial effects observed from LI-rTMS observed in our animal model can be observed in all tinnitus patients.

Lastly, another difference between our study and human studies is that the LI-rTMS used in our study is different from the high intensity rTMS applied in humans because it does not induce action potentials and therefore is unlikely to induce classical forms of synaptic plasticity such as LTD. Rather LI-rTMS induces changes in intracellular calcium levels and regulates gene expression^64^, which underpin short and long lasting changes in excitability^65^,^66^ and structural reorganisation^16^. Such long-lasting changes are in line with our findings that the effects of LI-rTMS on behaviour could still be detected at three days after the final LI-rTMS session. Our results suggest that reducing the intensity of magnetic field stimulation from the conventional high intensity to subthreshold levels may be a promising avenue for tinnitus therapy.

### Spontaneous activity

It is well established from human studies that tinnitus is strongly associated with abnormal levels of activity in the brain of patients^9^,^55^,^56^,^67^,^68^,^69^. This finding has been replicated in animal models of hearing loss and tinnitus which show abnormally high levels of spontaneous activity in the main nuclei of the auditory pathway^10^,^70^,^71^,^72^,^73^. A relationship between elevated spontaneous activity and tinnitus is further supported by firstly the observations in humans that the frequency of hearing loss often corresponds to the perceived pitch of tinnitus^74^ and secondly, the findings in animals that the frequency region of hearing loss is associated with abnormal activity in tonotopically corresponding frequency ranges of central auditory structures^10^,^33^,^48^,^75^. This was also confirmed in the present data showing higher spontaneous activity in the frequency region ≥10 kHz compared to the region with CFs < 10 kHz.

It may be possible that in our study the LI-rTMS treatment has lowered abnormal levels of activity in auditory cortex, thereby reducing the perception of tinnitus. In a recent study, the same intensity of LI-rTMS modulated motor cortex excitability in rats^39^. In addition, the small reduction of increased spontaneous activity in the IC, though not statistically significant, may have been the result of activation of descending pathways originating in the auditory cortex. The auditory cortex is capable of modulating the IC and there are extensive anatomical studies demonstrating direct descending projections from the auditory cortex onto the IC^76^,^77^,^78^. These projections play a role in auditory plasticity and can change the sensitivity of IC neurons to sound frequency, intensity, duration and location^79^,^80^,^81^. In addition, the effects on the IC can be excitatory or inhibitory^82^,^83^. Alternatively, we cannot rule out the possibility that LI-rTMS directly affected the IC, but this is unlikely because the field strength decreases exponentially from the coil and would be negligible in this deeper region.

The fact that tinnitus attenuation is observed without an effect on hyperactivity in IC may seem contradictory to the notion that hyperactivity is involved in the generation of tinnitus. However, in would be in line with the thalamic gating hypothesis or noise cancellation network proposed by Rauschecker^84^. According to this hypothesis hearing loss would always lead to hyperactivity in the auditory pathway but this would generally be gated at the level of the thalamus. A failure in this gating mechanism would lead to the hyperactivity being propagated to the cortex leading to tinnitus perception. The rTMS treatment may have affected thalamocortical processing, thereby decreasing the hyperactivity at the thalamic and cortical level, and thus tinnitus perception, while leaving unaltered the hyperactivity at the lower levels of the auditory cortex such as in the IC.

### BDNF

The LI-rTMS treatment did not affect BDNF levels in IC or auditory cortex and so this molecule does not seem to be involved in the mechanism by which LI-rTMS affects tinnitus. This was surprising as LI-rTMS has previously been shown to upregulate BDNF in visual brain regions^16^,^17^ in intact mice, but not in the retina following injury^85^. Our finding that low-frequency LI-rTMS does not strongly affect BDNF levels in auditory structures in a guinea pig model of tinnitus suggests that LI-rTMS effects may be brain-region specific and/or dependent on the presence of injury. However, we cannot rule out transient effects of LI-rTMS on BDNF, either at earlier time-points, or within a specific window post-stimulation, as shown previously in the mouse visual system, though using a different stimulation protocol^16^,^17^.

As BDNF levels were higher in the right (contralateral) IC than in the left IC, regardless of receiving rTMS or sham, our results suggest acoustic trauma itself may affect BDNF, suggesting possible BDNF involvement in the generation of tinnitus. We have previously shown that BDNF levels are significantly elevated in the IC two weeks after a mechanical, but not after an acoustic trauma (compared to sham-trauma animals)^86^ though it should be noted that other studies have found increased BDNF levels in IC after acoustic trauma^87^. The present data suggest ongoing changes in BDNF levels at longer recovery times after the acoustic trauma.

### Cochlea

Finally, it is theoretically possible that LI-rTMS would affect tinnitus via the cochlea. During the acoustic trauma, the right ear was blocked for protection and therefore CAP thresholds were expected to be unaffected. However, some of the LI-rTMS treated animals showed abnormal elevation of thresholds recorded from their right cochlea (see Fig. 2D). As the target of LI-rTMS was the right auditory cortex and because the right cochlear thresholds were recorded only at the final electrophysiological experiment, possible detrimental effects of LI-rTMS on cochlear thresholds cannot be ruled out. However, there are no known reports in humans of detrimental effects of rTMS to hearing and because only some of the animals showed these elevations of the right cochlear thresholds, these elevations may already have been present before LI-rTMS was applied. Secondly, since the results showed a deterioration of the cochlear threshold, one would expect a deleterious effect on tinnitus not an alleviation of the effects. Finally, our human and animal bioassay of the intensity of the clicks produced by the small coil indicated that the sound intensity level of the clicks is very low (approximately 20 dB above threshold), which is unlikely to cause any permanent damage.

## Methods

### Animals

Nineteen pigmented guinea pigs were used. All guinea pigs were adults, with 8 females and 11 males weighing between 320 and 640 g (mean 428, SEM 7 g) at the time of acoustic trauma. Guinea pigs were bred at the University of Western Australia and housed at the Preclinical Facility, University of Western Australia. Animals were group housed (max. 2 per cage) in standard cages (L 55 cm; W 38 cm; H 26 cm), on a 12 hr light-dark cycle and controlled temperature (22°, SEM 2 °C), with food and water available *ad libitum*. All experimental protocols conformed to the Code of Practice of the National Health and Medical Research Council of Australia and were approved by the Animal Ethics Committee of The University of Western Australia.

### Experimental design

**Figure 5:**
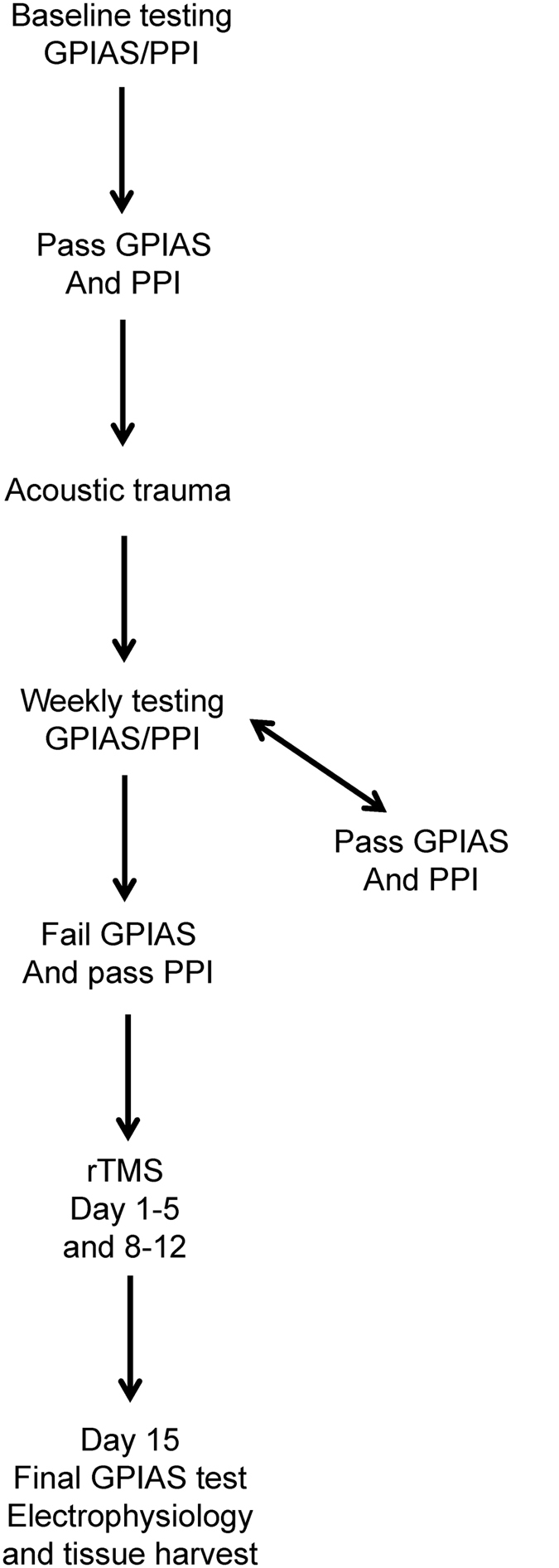
Flow diagram of experimental design. Animals were tested for GPIAS and PPI to obtain baseline measures. When animals passed both tests (see text for criteria applied) they underwent an acoustic trauma. After acoustic trauma weekly testing of GPIAS and PPI commenced. When animals passed weekly testing continued, when animals failed GPIAS twice but passed PPI they were deemed to show behavioural evidence of tinnitus and the first Monday after this occurred, rTMS treatment commenced (2 weeks Monday to Friday). On the Monday after these 2 weeks animals were put through a final GPIAS test and underwent a final electrophysiological experiment during which the spontaneous firing rates in IC were recorded. At the end of this experiment animals were euthanized and tissue for the auditory cortices and IC collected.

The experimental design and timeline is shown in Fig. 5. Guinea pigs were first tested to obtain baseline measures for behavioural outcomes (gap prepulse inhibition of acoustic startle (GPIAS) and Prepulse Inhibition (PPI), described below) and then exposed to a unilateral acoustic trauma (left ear). After trauma, animals underwent weekly behavioural tests (GPIAS and PPI) to assess for tinnitus. Once it was established that the animals had tinnitus, the animals underwent LI-rTMS or sham treatment for 10 minutes daily for 10 working days (Monday to Friday) in a 14 day period. On the 15^th^ day after commencing treatment, animals underwent GPIAS testing again.

On that same day or on the day after, animals underwent non-recovery electrophysiological (single-neuron) recordings in the inferior colliculus to measure spontaneous firing rates. Animals were euthanized and left and right inferior colliculi and auditory cortices collected for analysis of BDNF expression. Animals that did not develop tinnitus did not proceed in the study.

### LI-rTMS parameters

The small animal coil (inner diameter 4 mm; outer diameter 8 mm) was constructed by winding insulated copper wire (0.125 mm diameter, Brocott UK, United Kingdom; 780 turns) around a plastic bobbin using a fine wire coil-winding machine (Shining Sun SW-202B, China). Stimulation parameters were controlled by a waveform generator (Agilent 335141B, USA) connected to a bipolar voltage programmable power supply (KEPCO BOP 100–4 M, USA). Experiments were conducted at 100% of the maximum power supply output (±100 V) using custom biphasic waveforms (400 μs rise time) (Agilent Benchlink Waveform Builder, USA).

A Hall Effect probe was used to measure magnetic field intensity delivered by the coil (Honeywell SS94A2D, USA). Intensity at the base of the coil was 90 mT and the half-maximum field occurred at 2 mm_z axis_, 3.5 mm_xy axis_, defining a focal stimulation area that was restricted to the auditory cortex of one hemisphere. These intensities have previously been shown to modulate motor cortical excitability in rats^39^.

During LI-rTMS or sham treatment animals were awake and held gently but securely on the experimenter’s lap and the coil was placed over the right auditory cortex with the central axis perpendicular to the surface of the skull (contralateral to the acoustic trauma or sham surgery ear) contacting the skin. Animals showed no stress and remained still throughout sessions. We confirmed that the coil did not generate heat above ambient temperature by attaching the coil to a K-type thermocouple (−40° to 260 °C, Dick Smith Electronics Q1437, Australia) and recording temperature every 50 pulses. In addition, our LI-rTMS protocol did not trigger any muscle twitches in the animal. For these reasons, we used the coil switched off as a sham treatment to control for handling.

The coil when turned on does produce a just audible sound. Attempts were therefore made to measure the sound intensity of the brief clicks emitted by the TMS coils using three different methods. Firstly, a ½” condensor microphone (Bruel and Kjaer Type 4134) was placed as close as possible to the coil. The microphone was calibrated using a Bruel and Kjaer Type 4231 calibrator. The output of the ½” microphone was viewed directly on an oscilloscope screen. It was found that there was a major artifact in the microphone output that was induced by the magnetic field from the coil and this could not be eliminated by shielding. This induced artifact was critically dependent on the spatial relationship between the TMS coil and the recording microphone, with the smallest artifact being present when these were at right angles to each other. Under these circumstances, the signal from the microphone (presumably a mixture of induced artifact and real acoustic signal) had a peak amplitude that corresponded to approximately 75 dB SPL (re 20 μPa). Because there was no way of separating artifact and acoustic signal in this method, this is likely an overestimate of the real sound pressure of the acoustic clicks emitted by the coil. Our second method to measure the sound intensity of the clicks emitted by the rTMS coil was a human bioassay method, using two normal hearing human listeners. The sound from the coil inserted into the external ear canal of one ear was matched in apparent loudness to a brief click presented to the other ear using calibrated custom sound generating equipment described in detail elsewhere^10^. The duration and spectral content of the clicks were adjusted to match as closely as possible the clicks emitted by the TMS coil. This method yielded an estimate of the peak intensity of the TMS clicks of approximately 26 dB SPL. Thirdly, we attempted to measure the intensity of the clicks by measuring the auditory nerve response in one guinea pig in response to activation of the coil (animal bioassay). For this purpose the animal was anaesthetized and surgery performed to record the compound action potential (CAP) of the auditory nerve (method described in detail below in section “Single neuron recordings of spontaneous firing”). LI-rTMS stimulation was then applied to the auditory cortex ipsi- or contralaterally to the side of CAP recording and the amplitude of the CAP was measured (N1-P1 peak amplitude). This CAP amplitude was then matched to the CAP amplitude in response to a short noise burst (0.5 ms) of a specific intensity presented to the side of recording. LI-rTMS applied contralateral to the recorded cochlea did not result in a recordable CAP. LI-rTMS applied ipsilateral to the recorded cochlea did result in a detectable CAP, with an amplitude which was equivalent a noise burst 20 dB above threshold.

### Behavioural testing for tinnitus

Behavioural testing for tinnitus consisted of gap prepulse inhibition of acoustic startle (GPIAS) and Prepulse Inhibition (PPI) and was performed as described previously^32^. PPI occurs when a weaker pre-stimulus, or prepulse, inhibits the reaction to a stronger stimulus. GPIAS is a variation of PPI and consists of a comparison between two conditions. Both conditions consist of a background noise and startle pulse which elicits a startle response. However, in one condition, there is a gap within the continuous background noise which precedes the startle pulse. The gap in this case works as a prepulse. In normal animals, this condition results in inhibition of the startle response. It is thought that animals experiencing tinnitus that is qualitatively similar to the background noise, show decreased startle inhibition, i.e. deficits of GPIAS, because the tinnitus “fills in” the gap in the background noise^37^. A deficit in the GPIAS test (p > 0.05 see below) could also be due to hearing loss (when an animal does not hear the background noise it cannot detect a gap therein) and therefore a PPI test is performed in parallel to the GPIAS test, using for the prepulse the same parameters as for the background noise in order to establish that the animal can hear the prepulse/background noise. Therefore, animals that fail GPIAS testing but pass PPI testing are thought to have tinnitus^32^,^43^.

During behavioural testing sessions, performed within a soundproof room, animals were mildly restrained in a clear Perspex holder, placed on top of a custom-designed force transducer. Up to 4 animals were tested simultaneously. Animals were left in the dark soundproof room for five minutes to allow habituation before measurements were obtained by custom written software (N. Yates). Startle stimulus (Radio Shack 401278B; 115 dB SPL, narrowband noise, centre frequency 1 kHz, bandwidth 100 Hz, 20 ms duration, 0.1 ms rise/fall time) was the same for all PPI and GPIAS tests. Acoustic stimuli were calibrated using a ½” microphone positioned at the location of the animal’s external ear canal. An additional speaker (Beyer DT 48) was used to administer a continuous background noise or a prepulse sound stimulus for GPIAS or PPI, respectively. Background noise/ prepulse consisted of a narrowband noise centred at either 8 or 14 kHz (3 dB bandwidths = 1 kHz) with an intensity of 70 dB SPL. Animals had to pass (see below) PPI once and GPIAS twice before an acoustic trauma was performed.

The PPI test consisted of 50 trials where a startle stimulus was presented. Half of these trials also featured a 50 ms prepulse presented 100 ms before the startle (prepulse trial), while the other half did not (no-prepulse trial). The order of prepulse and no-prepulse trials during a test was random and the intervals between startle stimulus presentations varied (15–30 s).

The GPIAS test was similar to the PPI, however involved a continuous background noise and a 50 ms gap (silence) instead of a prepulse. A GPIAS test consisted of 50 trials, with half of the trials including a gap preceding the startle stimulus by 100 ms (gap trial), while the other did not (no-gap trial). The order of gap and no-gap trials was random and intervals between startle stimulus presentations varied (15–30 s). One testing session consisted of 2 PPI or 2 GPIAS tests (1 with 8 kHz and 1 with 14 kHz background noise/prepulse). The order of using 8 and 14 kHz background noise/prepulse was alternated between sessions. There was at least one day between testing sessions.

The startle response generated by the animals was recorded by a force transducer and processed by custom written software in LabView (N. Yates) to calculate acoustic startle response as the ratio between the root mean square (RMS) of the force produced during the startle response and the RMS of baseline force for each trial. The startle response ratios were compared between the two trial conditions (gap vs. no-gap for GPIAS tests and prepulse vs. no-prepulse for PPI tests) in each test using a t-test. A statistically significant difference (p < 0.05) between trial conditions indicated a “pass” for that test. The animal was deemed to have failed the test when there was no significant difference between the gap and no gap condition (GPIAS test) or between the prepulse and no prepulse conditions (PPI test) (p > 0.05). The first 4/50 trials were not included to avoid habituation bias^32^,^43^. For each test the average “suppression” of the startle reflex caused by the pre-startle gap (or prepulse) was calculated and converted to a percentage.

Once animals passed the GPIAS test twice and a PPI test, they were exposed to acoustic trauma. After acoustic trauma, weekly GPIAS testing resumed. When an animal passed the GPIAS test the weekly testing continued. When an animal failed the GPIAS testing (i.e. no significant GPIAS), a PPI test was performed. If the animal passed the PPI test (p < 0.05) then two days later another GPIAS test was performed. If the animal failed this again, it was deemed to have tinnitus and commenced LI-rTMS or sham treatment on the first Monday after.

### Acoustic trauma

Acoustic trauma procedures were similar to those described in previous studies from our laboratory^10^,^30^,^31^. For acoustic trauma, animals received a subcutaneous (s.c.) injection of 0.1 ml Atropine (0.6 mg/ml) followed by an intraperitoneal (i.p.) injection of Diazepam (5 mg/kg). Twenty minutes later, intramuscular (i.m.) Hypnorm (0.315 mg/ml fentanyl citrate and 10 mg/ml fluanisone; 1 ml/kg) was administered. Once foot withdrawal was absent, the animal was placed on a heating blanket and mounted into hollow ear bars in a soundproof room. If foot withdrawal returned during the experiment a 1/3 top-up dosage of Hypnorm i.m. was administered. Eye drops were applied to the eyes and 0.1 ml Lignocaine (20 mg/ml lignocaine hydrogen chloride) injected s.c. to where the incision was to be made. The bulla was then exposed and a small opening made in the bulla to enable placement of an insulated silver wire onto the round window to record a compound action potential (CAP) audiogram for frequencies ranging from 4 to 24 kHz^88^. Sound stimuli were presented in a calibrated closed sound system through a ½” condenser microphone driven in reverse as a speaker (Bruel and Kjaer, type 4134). Pure tone stimuli (10 ms duration, 1 ms rise/fall times) were synthesized by a computer equipped with a DIGI 96 soundcard connected to an analog/digital interface (ADI-9 DS, RME Intelligent Audio Solution). Sample rate was 96 kHz. The interface was driven by a custom-made computer program (Neurosound, MI Lloyd), which was also used to collect single neuron data during the final experiments. CAP signals were amplified (1000x), filtered (100 Hz–3 kHz bandpass) and recorded with a second data acquisition system (Powerlab 4SP, AD Instruments). The right ear was then blocked, while the left ear exposed to a continuous pure tone of 10 kHz at 124 dB for 2 hours. Afterwards, the audiogram was measured once more to determine the extent of acute hearing loss, the incision was sutured and animals were allowed to recover from anaesthesia.

### Single neuron recordings of spontaneous firing

Procedures were similar to those previously described^10^,^48^. Animals were anaesthetised by s.c. administration of 0.1 ml atropine (0.65 mg/ml Atropine sulphate), followed by an i.p. injection of 30 mg/kg of sodium pentobarbitone. After 10 minutes, an i.m. injection of 0.15 ml of Hypnorm (0.135 mg/ml Fentanyl citrate, 10 mg/ml Fluanisone) was administered. Lignocaine (20 mg/ml) was administered s.c. to the incision areas. Once full surgical anaesthesia was achieved a tracheotomy was performed and the animal was artificially ventilated with carbogen (95% oxygen and 5% carbon dioxide). A full dose of the original Hypnorm dose was administered each hour, and half the pentobarbitone dose was administered every two hours to maintain surgical anaesthesia. 0.1 ml of Pancuronium (2 mg/ml Pancuronium bromide) was administered prior to single neuron recordings to induce paralysis. An electrocardiogram was monitored continuously throughout the experiment to ensure depth of anaesthesia. After the tracheotomy, animals were placed on a heating pad in a sound-proof room and mounted in hollow earbars. CAP audiograms were measured from both ears in the same manner as during the acoustic trauma procedure. A small craniotomy was performed exposing the cortex overlying the inferior colliculus and a tungsten-in-glass microelectrode was inserted into the cortex until the recorded electrical activity was indicative of the CNIC. The dorsal aspect of the CNIC was indicated by strong sound-driven activity with a short latency (cluster onset latencies < 6.5 ms) and a systematic progression from low to high characteristic frequencies (CF) with increasing depth. The craniotomy was covered with a 5% agar solution to prevent dehydration and ensure stability of recording. When a CNIC neuron was isolated, its CF and threshold at CF were determined audio-visually and depth from the cortical surface was recorded using methods described previously^52^,^89^. Spontaneous activity was measured during a 10 s sample period.

### BDNF expression

At completion of recording, animals received an overdose of 0.3 ml of Lethabarb (325 mg/ml pentobarbitone sodium) and were decapitated using a guillotine. Brains were quickly dissected and placed in a petri dish containing ice cold saline. Using the rhinal sulcus as a guide, left and right auditory cortices and inferior colliculi samples were rapidly excised. Samples were dry stored at −80 °C until homogenized by mechanical disruption in 1 mL lysis buffer (100 mM PIPES pH 7, 500 mM NaCl, 0.2% Triton X-100, 0.1% sodium azide, 2 mM EDTA, mini protease inhibitor tablets (Roche Biochemicals, Indiana USA; 1 tablet added per 10 mL buffer^90^) and centrifuged at 3823 × *g* for 1 h at 4 °C. Total protein content was determined using the BCA method according to kit instructions (Pierce™ BCA Protein Assay Kit, Thermo Fisher Scientific™, Illinois USA) on samples diluted 1:10 with PBS so that protein content was within the assay range. The supernatant was analyzed by ELISA for BDNF according to the manufacturer’s instructions (ChemiKine BDNF Sandwich ELISA; Millipore Bioscience Research Reagents, Darmstadt, Germany). Optical absorbance measures (450 nm) were performed automatically using a PerkinElmer 2300 spectrophotometer plate reader with EnSpire software. BDNF concentrations were interpolated from the standard curve (pg/mL) and calculated as percentage of total protein for each sample. All biochemical procedures were performed blinded to stimulation condition.

### Statistical analysis

For further analysis of the spontaneous firing rates data, we converted the audiovisual CF into a nominal CF. For this purpose the recorded neuron’s electrode depth into the CNIC (from cortical surface) was converted into frequency (for detailed method and validation see also^10^,^48^ because the peripheral threshold loss can affect the CF based on the lowest threshold of tuning curves in the damaged region. All data shown arises from nominal CF calculations.

Spontaneous firing rate data are not normally distributed. Therefore, to test for statistically significant differences in overall spontaneous firing rates between the sham and rTMS groups a two-tailed Mann-Whitney U test was used. When comparing the different frequency regions in IC between the two treatment groups, we used a Kruskall-Wallis test with a Dunn’s correction for multiple comparisons. We used a two-way mixed ANOVA to compare LI-rTMS and sham on CAP threshold changes at each frequency (repeated measures factor). BDNF concentrations as a percentage of total protein were analysed separately for the auditory cortex and inferior colliculus, using a two-way mixed ANOVA to compare stimulation condition (sham, rTMS), including side (stimulated, non-stimulated) as repeated measures factor. Results were considered statistically significant if p < 0.05. Statistics were performed using Graphpad Prism (version 6).

## Additional Information

**How to cite this article**: Mulders, W. H. A. M. *et al*. The effects of repetitive transcranial magnetic stimulation in an animal model of tinnitus. *Sci. Rep.* **6**, 38234; doi: 10.1038/srep38234 (2016).

**Publisher’s note:** Springer Nature remains neutral with regard to jurisdictional claims in published maps and institutional affiliations.
